# The Role of Neutrophil Extracellular Traps (NETs) in the Pathogenesis of Systemic Lupus Erythematosus and Antiphospholipid Syndrome

**DOI:** 10.3390/ijms241713581

**Published:** 2023-09-01

**Authors:** Tatiana Reshetnyak, Kamila Nurbaeva

**Affiliations:** Department of Thromboinflammation, V.A. Nasonova Research Institute of Rheumatology, 115522 Moscow, Russia; sokrat@irramn.ru

## 1. Introduction

Systemic lupus erythematosus (SLE) is a systemic autoimmune disease of unknown aetiology. It is characterized by impaired tolerance to a wide range of cellular and cytoplasmic components with the development of immunoinflammatory tissue damage [1]. Antiphospholipid syndrome (APS) is a systemic autoimmune disease characterized by recurrent vascular thrombosis of all calibers and localizations and/or obstetric morbidity and the obligatory detection of persistently positive antiphospholipid antibodies (aPL) [2]. Up to 50% of SLE patients have aPL [3], and 30–40% have combined SLE and APS [4]. The frequent combination of both diseases and common genetic, clinical and laboratory manifestations suggest common pathogenetic mechanisms [5]. Common links in the pathogenesis of SLE and APS may include activation of B cells with synthesis of autoantibodies directed against their own antigens [6,7], increased levels of circulating immune complexes [8,9,10], proinflammatory cytokines [11,12], complement activation [13,14], type I interferon hyperreactivity [15,16,17], mitochondrial dysfunction [18,19] and increased neutrophil extracellular trap (NET) formation [20,21].

Neutrophil extracellular traps (NETs) are web-like structures composed of modified chromatin, histones and proteins of neutrophil granules and cytoplasm, such as myeloperoxidase (MPO), neutrophil elastase (NE), LL-37, matrix metalloproteinases, cathepsin G and others [22]. NETs serve as a “trap”, limiting the spread of microorganisms and promoting their destruction [23]. However, the role of NETs is not limited to an antimicrobial protective function. The development and maintenance of many chronic non-communicable diseases are associated with excessive formation or impaired clearance of NETs [24]. NET formation by neutrophils is triggered by various stimuli, such as microbial antigens, immune complexes, antibodies, pro-inflammatory cytokines, complement components, etc. [25]. It is currently accepted that it is the stimulus that determines the further path of NETosis: classical (suicidal) or alternative (vital) NET formation [26]. The detailed mechanism of NET formation is considered in the following reviews [22,27]. In brief, during suicidal NETosis, the enzyme nicotinamide adenine dinucleotide phosphate oxidase (NADPH oxidase) is activated after the activation of neutrophils via a stimulating factor. This leads to the synthesis of reactive oxygen species (ROS). ROS production leads to the release of granular proteases into the cytoplasm and also activates the enzyme peptidyl arginine deiminase 4 (PAD4). PAD4 activates histone citrullination. The exposure of the granule proteins and the citrullination of histones leads to chromatin decondensation. The nuclear envelope disintegrates, and decondensed nuclear chromatin mixes with cytoplasmic and granular components. Subsequently, the plasma membrane ruptures, and NETs are released into the extracellular space. An alternative mechanism by which the neutrophil remains viable after NET release is called vital NETosis. During vital NETosis, neutrophils release NETs without the disruption of the nucleus, plasma membrane or activation of NADPH oxidase. It is also worth highlighting mitochondrial NETosis. This is associated with the release of mitochondrial DNA, which has been less studied.

**Figure 1:**
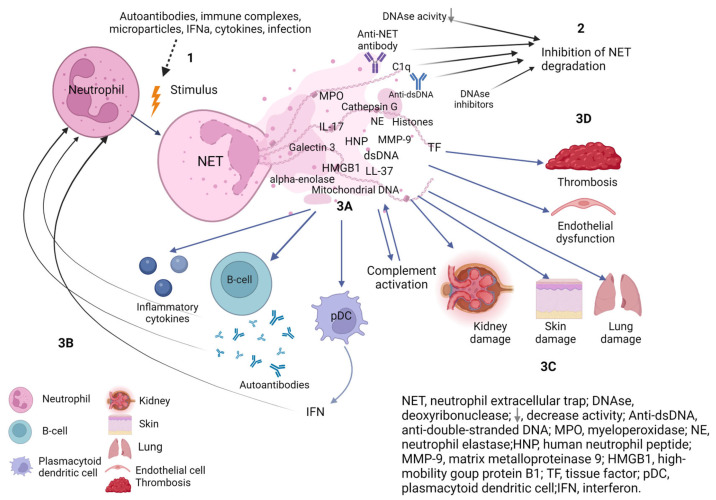
The role of NETs in the pathogenesis of systemic lupus erythematosus. (**1**) Different stimuli trigger neutrophil activation with increased NETs release. (**2**) Deficiency, decreased activity of DNAse or presence of DNAase inhibitors and factors protecting NETs from degradation (C1q, anti-NET antibodies, anti-dsDNA) prevent efficient NET clearance. Increased synthesis and/or impaired degradation of NETs contribute to NETs persistence in tissues and circulation. (**3A**) NETs components are immunogenic and activate B cells to synthesise autoantibodies and plasmacytoid dendritic cells to synthesise interferons and the complement system. (**3B**) Autoantibodies, proinflammatory cytokines, and IFN activate neutrophils to release NETs, which contribute to a vicious cycle of inflammation. (**3C**) The components of NETs cause damage to their own tissues with the development of endothelial dysfunction, damage and fibrosis of the kidney, lungs and skin. (**3D**) NETs components affect the hemostasis system and provide a scaffold for thrombus formation. Created with BioRender.com.

In SLE and APS, as in many other diseases, suicidal NETosis is the most studied [27]. NETs may be involved in the pathogenesis of SLE and APS because NETs are the sources of autoantigens [28], activate the complement system [29], induce the synthesis of interferon-alpha (IFNα) [30], activate immunoinflammatory cells [30], activate the blood coagulation system and contribute to the formation of blood clots [21], causing endothelial dysfunction and tissue damage [28]. There is much more data on the contribution of NETs to the pathogenesis of SLE than APS. However, in view of the common mechanisms of disease development, we present a possible scheme of the role of NETs in the development of SLE and, to a lesser extent, APS (Figure 1). Next, we review the studies that have investigated the contribution of NETs to the development of SLE and APS. It should be emphasised that the results are contradictory.

## 2. NETosis in SLE

### 2.1. NETs Formation in SLE

#### 2.1.1. Spontaneous and Stimulated Formation of NETs by Neutrophils of SLE Patients

Neutrophils from SLE patients were found to spontaneously release more NETs than neutrophils from healthy controls [28,31,32,33,34,35], similar to studies in animal models of SLE [36,37]. However, stimulated NETosis in SLE does not lead to a further increase in NET release [28]. These observations suggest that there are priming factors present in the blood of SLE patients that pre-stimulate neutrophils in vivo, leading to increased “spontaneous” formation of NETs, and subsequent depletion results in neutrophil resistance to further stimuli. Lande et al. [30] linked the increase in NETs in SLE to the circulation of antibodies against the antimicrobial peptide LL37 (anti-LL37) and human neutrophil peptides (anti-HNP). These antibodies targeted the NETs components LL37 and HNPs. They were able to activate neutrophils from healthy donors to release NETs. Another study [38] found that incubation of healthy donor neutrophils with anti-LL37 and anti-HNPs resulted in increased synthesis of NETs, whereas NETs were not released when neutrophils were incubated with control IgG. Gestermann et al. [39] found a significant correlation between the levels of antibodies to LL37 in peripheral blood and the number of neutrophils undergoing NETosis in patients with SLE. There is also evidence that other autoantibodies may be involved in the induction of NETosis. For example, anti-RNP (anti-ribonucleoprotein) IgG contributed to increased NET formation in SLE but not in healthy controls [32]. The RNP-anti-RNP complex stimulated control neutrophils to release more NETs than controls treated with RNP/IgG. The activation of NETosis was dependent on the activity of the inositol-requiring transmembrane kinase/endonuclease 1 alpha (IRE1 alpha), the mitochondrial ROC and caspase-2 [40]. In addition, immune complexes consisting of matrix metalloproteinase-9 (MMP-9) and anti-MMP-9 from the serum of SLE patients activated neutrophils to produce NETs [41]. The relationship between the increased ability of neutrophils to form NETs and the clinical and laboratory manifestations of SLE remains unclear. A pathological subset of neutrophils called low-density granulocytes (LDG) was found to have an increased ability to spontaneously form NETs in SLE by Villanueva et al. [28]. However, the authors did not find a significant correlation between the number of LDGs undergoing NETosis and the activity of SLE, the presence of autoantibodies or the titer of autoantibodies. In a study by Van der Linden et al. [42], neutrophils from healthy controls produced significantly more NETs upon exposure to plasma from patients with SLE ± APS than to plasma from controls. Plasma from 60% of patients with SLE and 61% of patients with SLE + APS induced NETs release compared to 7% of controls. In addition, compared with patients whose plasma induced low NETs release, patients with high NETs release had higher levels of antibodies against dsDNA and interferon-stimulated gene (ISG) expression. However, NET formation did not correlate with SLE activity or the presence of active lupus nephritis. Nakazawa et al. [43] also found no association between SLE activity and NET formation. Conversely, serum from patients with high SLE activity induced more NET formation than serum from patients with low disease activity, according to van Dam et al. [44]. In addition, the depletion of IgG resulted in a significant decrease in NETosis in SLE. The authors concluded that immune complexes are responsible for NET formation. El-Ghoneimy et al. [45] found a direct correlation between NETs levels and the systemic lupus erythematosus disease activity index (SLEDAI), with kidney involvement scores on the SLEDAI, proteinuria, erythrocyte sedimentation rate (ESR) and anti-dsDNA, and an inverse correlation with complement component C3. Another study by Frangou et al. [35] showed that neutrophils from patients with a high level of SLE activity had an increased release of NET compared to neutrophils from patients with a low level of SLE activity or compared to control neutrophils. However, contrary to previous authors, Bruschi et al. [46] did not find increased spontaneous or induced NETosis by neutrophils from SLE patients compared to controls.

#### 2.1.2. Peptidyl Arginine Deiminase 4 (PAD4) in SLE

PAD4 is an enzyme required for histone citrullination during NETosis. Through the action of PAD4, positively charged arginine is converted to citrulline, leading to chromatin decondensation by weakening the link between histones and negatively charged DNA [47]. There are still conflicting data regarding the role of PAD4 in NET formation in SLE. In the study by Knight et al. [36] using a mouse model of SLE (NZM mice), the PAD inhibitor chloramidine blocked the formation of NETs and resulted in a decrease in complement levels, autoantibodies, IgG deposition in the kidneys, endothelial dysfunction and delayed development of arterial thrombosis. In another mouse model of SLE (MRL/lpr mice), the in vitro inhibition of PAD by Cl- amidine and BB-Cl- amidine led to a reduction in the formation of NETs. The treatment of MRL/lpr mice with Cl-amidine and BB-Cl-amidine for 6 weeks significantly reduced both spontaneous and induced NET formation ex vivo. Clinically, PAD4 inhibition resulted in a reduction in endothelial dysfunction, type I IFN gene expression and suppression of inflammation in the kidney and skin [37].

Neutrophils from PAD4-deficient (PAD4−/−) mice synthesised fewer NETs in response to stimulation than control neutrophils in a study by Liu et al. [48]. PAD4−/− mice had significantly lower levels of autoantibodies, type I IFNs, when exposed to the Toll-like receptor 7 (TLR-7) agonist imiquimod (imiquimod-induced lupus model) compared to mice with functional PAD4. The authors also showed that compared to wild-type mice in which imiquimod induced the development of SLE-like disease, PAD4−/− mice exposed to imiquimod had significantly less IgG deposition in the kidneys, endothelial dysfunction and lupus-like symptoms. In another study [49], in an imiquimod-induced lupus model, PAD4 knockout (−/−) resulted in the complete disappearance of spontaneous NETosis, whereas imiquimod stimulated wild-type mice to form NETs. Clinically, PAD4 (−/−) mice had significantly reduced proteinuria and splenomegaly and histologically showed reduced neutrophil infiltration of the kidneys and skin compared to wild-type mice receiving imiquimod. However, anti-dsDNA antibody antibodies levels did not differ between the two mouse strains. In a mouse model of SLE (Fcgr2b−/−), blockade of the PAD4 enzyme after exposure to ischaemia-reperfusion renal injury also resulted in a decreased NET formation, decreased blood levels of antibodies to dsDNA, and manifestations of lupus nephritis [50]. Conversely, there are conflicting data that suggest a neutral or even protective role of PAD4 in the development of SLE. Pristane-induced lupus was exacerbated in PAD4-deficient mice with reduced NET formation. PAD4 (−/−) mice had increased levels of circulating autoantibodies, proinflammatory cytokines and symptoms of glomerulonephritis [31]. In a study by Gordon et al. [51], there was no difference in the incidence of SLE-like disease in PAD4 (−/−) and PAD4 (+/+) MRL/lpr mice. In addition, the inhibition of PAD4 by chloramidine did not affect the degree of kidney damage in a passive model of lupus nephritis induced by the administration of serum from SLE patients.

#### 2.1.3. NOX-2 NADPH-Oxidase in SLE

NADPH oxidase catalyses the transfer of electrons from NADPH to molecular oxygen for the generation of reactive oxygen species (ROS). The activation of NADPH oxidase is required for the formation of NETs in the classical NADPH-dependent NETosis [22]. Pharmacological inhibition of NADPH oxidase activity blocked the release of NETs from neutrophils stimulated with anti-LL37 in vitro [38]. Similarly, the in vitro release of NETs induced by anti-RNP antibodies [32] or by MMP-9-containing immune complexes [41] was dependent on ROS.

However, a paradoxical role of NADPH oxidase as a protective factor in SLE has been observed in vivo. In a mouse model of SLE (MRL/lpr), the genetic deficiency of NADPH oxidase not only failed to suppress disease progression but also caused an increase in lupus-like symptoms [52]. Kienhöfer et al. [31] also found that, in a pristane-induced model of SLE, a deficient mutation in the NADPH oxidase gene led to increased production of autoantibodies, proinflammatory cytokines, activation of the complement system, interferon gene expression, exacerbation of glomerulonephritis and worsened survival. The administration of an NADPH oxidase activator to wild-type mice resulted in the attenuation of pristane-induced SLE, as evidenced by a reduction in proteinuria, deposition of immune complexes in the kidneys and decreased autoantibody levels.

In a study by El-Ghoneimy et al. [45], NADPH oxidase activity in patients with SLE, as assessed by the dihydrorhodamine test (DHR), did not differ between patients and healthy controls. At the same time, NET formation by neutrophils was significantly higher in patients with SLE compared to controls. There was a significant negative correlation between NETs and DHR levels. The authors concluded that NETosis in their cohort was predominantly mediated via an NADPH-independent pathway. Clinically, elevated NETs were associated with SLE exacerbation and lupus nephritis activity, whereas NADPH oxidase activity was associated with remission.

A possible explanation for the exacerbation of SLE symptoms is the pleiotropic immunological action of NADPH oxidase and ROS, which may exert anti-inflammatory effects [53], as well as the activation of NADPH-independent mitochondria-associated NETosis. Indeed, patients with chronic granulomatous disease (CGD) who are genetically deficient in NADPH oxidase have an increased risk of developing autoimmune diseases, including SLE. Lood et al. [54] showed that the increased production of mitochondrial ROS and activation of NADPH-independent NETosis in CGD may be a compensatory mechanism associated with the absence of NADPH oxidase function.

#### 2.1.4. Mitochondria and NETs

Mitochondria can participate in NETosis by generating reactive oxygen species (ROS) and releasing mitochondrial DNA (mtDNA) [40,44,54,55,56].

##### Mitochondrial ROS (mtROS)

There is an NADPH-independent NETosis that is realised under the action of mitochondrial ROS. Lood et al. [54] demonstrated the role of mtROS in the development of NETosis and the pathogenesis of SLE. Mitochondrial ROS production was increased in low-density granulocytes (LDGs) from patients with SLE compared to healthy controls or autologous lupus high-density neutrophils (HDN). Mitochondrial ROS were shown to be essential for NETosis, as the mitochondria-targeted antioxidant MitoTEMPO reduced spontaneous NETosis of LDGs. The investigators also found that SLE patients had significantly higher plasma levels of NETosis indicators compared to controls. Furthermore, in a mouse model of SLE (MRL/lpr), prophylactic treatment with MitoTEMPO resulted in a reduction in spontaneous NETosis, IFNα release and proinflammatory cytokines. This was clinically manifested by reduced proteinuria, reduced renal immune complex deposition and reduced serum anti-dsDNA levels. Another study [55] also found that the treatment of MRL/lpr mice with a mitochondria-targeted antioxidant (MitoQ (200 µM)) resulted in reduced ROS and NET formation, deposition of immune complexes in the kidneys, improved renal function and reduced serum IFN-I levels without affecting autoantibody levels. The activation of the endoplasmic reticulum stress sensor IRE1α led to the generation of mtROS and the formation of NETs in a mouse model of SLE. The inhibition of IRE1α reduced mtROS and NETs formation in vitro, which was manifested in vivo by reduced splenomegaly and autoantibody levels [40].

##### Mitochondrial DNA (mtDNA)

The release of mitochondrial DNA into the extracellular space during NETosis was demonstrated by Wang et al. [56]. They found that NETs from SLE patients contained significantly more mtDNA than NETs from healthy controls. In addition, the amount of mtDNA in NETs was positively correlated with the SLEDAI. A study by Lood et al. [54] found that NETs spontaneously released by lupus neutrophils were enriched in mtDNA and 8-OH-deoxyguanosine (8-OHdG)-modified DNA compared to NETs induced by other stimuli in healthy control neutrophils. These NETs from SLE patients had a pronounced proinflammatory and interferogenic potential. Van Dam et al. [44] also found that NETs in SLE are enriched in oxidized mtDNA.

### 2.2. NETs Degradation in SLE

In addition to increased formation, impaired degradation of NETs is another cause of high levels of NETs. DNAase is an enzyme that cleaves single- and double-stranded DNA and maintains low levels of circulating free DNA. Since DNA is the major component of NETs, DNAases are thought to be the major structures responsible for cleaving NETs [57]. DNAases have been shown to degrade NETs in vitro and in vivo. A lack or reduction in DNase activity is known to contribute to the development of SLE in humans [58,59,60,61] and SLE-like disease in mice [62,63].

The association between DNase 1 and NETs in SLE patients was shown by Hakkim et al. [64]. They showed that in vitro destruction of NETs was reduced in SLE patients compared to healthy controls. The addition of exogenous DNase did not restore the degradation of NETs, leading the authors to conclude that SLE patients have DNase 1 inhibitors and/or factors that protect NETs from degradation. To test this hypothesis, MNase enzyme was added to patients’ serum. In some patients (group 1), the addition of MNase restored the degradation of NETs, indicating the presence of DNase1 inhibitors. In group 2, the addition of MNase resulted in only a slight increase in NETs degradation, suggesting the presence of factors that protect NETs from enzymatic degradation. The authors discovered that the serum of group 2 patients contained antibodies against NETs (anti-NET antibodies), which blocked the access of nucleases to NETs. Clinically, patients with impaired NET degradation, whatever the cause, were more likely to have lupus nephritis and higher levels of anti-dsDNA antibodies and antinuclear antibodies (ANA).

In an independent cohort, Leffler et al. [29] also found impaired NETs clearance in patients with SLE compared to healthy controls. In addition, the authors found that NETs clearance depended on SLE activity. Patients with high SLE activity had lower NET clearance than patients in remission. They also found, like the previous authors, that patients with impaired NETs degradation were more likely to have lupus nephritis, dsDNA antibody positivity, high anti-NET antibody levels and hypocomplementemia. In a prospective study, Leffler et al. [65] also found a decrease in NET clearance in the majority of patients with SLE. Reduced NET clearance in their cohort was associated with the presence of active glomerulonephritis, fever, alopecia, and increased antibody levels to dsDNA and histones. In another study, Leffler et al. [66] identified a cluster of SLE patients with reduced chromatin degradation regardless of the DNA source (including DNA NETs). Lupus nephritis and anti-dsDNA positivity were more common in this group than in other clusters with near-normal DNA degradation. Notably, the authors found no differences in DNAase 1 activity between patients and healthy controls. In contrast to the previous study, Nakazawa et al. [43] in another cohort of SLE patients found impaired NETs degradation due to decreased DNase 1 activity, which was not associated with SLE activity. Zhang et al. [67] also found a decrease in DNase activity in SLE patients, independent of disease activity or renal involvement. However, other authors found an association between decreased DNase activity and lupus nephritis [46,68]. In a study by Rother et al. [68], DNAase activity was found to be lower in patients with lupus nephritis than in patients without renal involvement. Bruschi et al. [46] also found that the activity of the enzyme DNAase was significantly lower in patients with SLE than in healthy controls. The lowest activity was found in patients with lupus nephritis. The serum levels of DNAse 1 and DNAse L3 did not differ between patients and healthy controls and did not correlate with DNAase activity. The authors concluded that DNase activity was reduced in patients due to the presence of inhibitory factors. All patients with DNase activity less than 30% had high levels of a marker of NETosis, the MPO-DNA complex, in the blood, indicating NETosis in vivo.

### 2.3. NETs in SLE

NET persistence due to increased NET formation and/or impaired NET clearance leads to various immunological abnormalities as NET components affect various cells of the adaptive and innate immune system and contribute to tissue damage in SLE.

#### 2.3.1. NETs and B-Cells

NETosis stimulates autoimmunity because the DNA, histones and proteins released by NETs are used as autoantigens. It is of great interest to study the protein composition of NETs and the potential importance of NETs as a source of post-translationally modified proteins in addition to DNA. Furthermore, it is important to understand their role as autoantigens. Bruschi et al. [69] investigated the proteomic composition of NETs from SLE patients. Many proteins found that NETs from SLE patients exhibited one or more post-translational modifications, such as oxidation, citrullination, phosphorylation, etc. In addition, the authors found that NETs from lupus nephritis patients had distinct protein compositional features that set them apart from other SLE patients. Fifteen proteins, including cytosolic, nuclear, membrane and organelle proteins, were found to distinguish SLE patients based on the presence of nephritis. One of these proteins is methyl-oxidised α-enolase, whose levels were significantly higher in NETs from patients with lupus nephritis. As anti-α-enolase antibodies are nephritogenic autoantibodies [70], Bruschi et al. [71] suggested that the DNA-α-enolase complex in NET activates B cells via TLR9 to produce antibodies against dsDNA and antibodies against α-enolase IgG2. In another study [44], NETs from SLE patients were found to contain high levels of high-mobility group box 1 (HMGB1) and neutrophil elastase. HMGB-1 facilitates endosomal uptake of self-DNA and activates B cells to synthesise antibodies against dsDNA. Lande and colleagues [30] also demonstrated that components of NETs activate B cells for autoantibody production. Neutrophil granular proteins of NETs form complexes with DNA (DNA/NET). These complexes lead to B cell activation via Toll-like receptor 9 (TLR9) and B cell receptor (BCR). Activated B cells synthesise autoantibodies against the antimicrobial NETs peptides LL37 and HNP, which in turn can induce the release of NETs by neutrophils in SLE patients, creating a vicious cycle of inflammation. In addition, the levels of antibodies to LL37 were directly correlated with the levels of antibodies to dsDNA and IFN-α in SLE patients. Gestermann et al. [39] also found that the LL37-DNA complex triggered the polyclonal activation of B cells with IgG production via TLR9. As in the previous study, anti-LL37 induced the formation of NETs. Other NET proteins, such as lactoferrin and lysozyme, were also found to form complexes with DNA that activated B cells. MMP-9 [41] and galectin-3 [33] of NETs also appear to stimulate B cells to produce autoantibodies to MMP-9 and antibodies to galectin-3. The circulating levels of these autoantibodies in the serum of SLE patients were significantly higher compared to controls.

#### 2.3.2. NETs and IFN

Self-nucleic acids as part of immune complexes can activate plasmacytoid dendritic cells (pDC) to produce type I IFN. For example, Lande et al. [30] found that the DNA-LL37 complex in SLE patients could activate pDCs via TLR9 to produce IFN-α. IFN-α production was significantly increased when anti-LL37, anti-HNP or anti-dsDNA, but not irrelevant antibodies, were added. Garcia-Romo et al. [32] showed that NETs induced by anti-RNP70 are potent activators of pDC in SLE. Activated pDCs synthesise IFN-α and the proinflammatory cytokines TNF-α and IL-6. SLE patients with reduced NET degradation had higher type I IFN activity compared to patients with efficient NET degradation [29]. SLE patients with high IFN activity had higher levels of LDGs and NET release than patients with low IFN [42]. In addition, it has been shown in murine models of SLE that blockade of NET formation by PAD4 [37,48] or inhibition of mitochondrial ROS [54,55] suppresses type I IFN synthesis. The separate role of mitochondrial ROS NETs in stimulating type I IFN formation is supported by Fortner et al. [55], who found that inhibition of mtROS led to a decrease in type I IFN levels independent of autoantibodies and immune complexes.

#### 2.3.3. NETs and Complement System

In vitro, NETs have been shown to activate the complement system. In vivo, Leffler et al. [29] found that SLE patients with low NET degradation had significantly lower serum levels of complement components C3 and C4. In addition, complement components themselves influence NETs. Leffler et al. found that the addition of C1q reduced the destruction of NETs due to the ability of C1q to bind to DNA and directly inhibit DNAase I. The inhibition of complement activation contributed to the reduction of NETs formation in vitro. In a study by Reshetnyak et al. [72], high levels of the MPO-DNA complex in the serum were associated with low levels of complement components C3 and C4.

#### 2.3.4. NETs and Tissue Damage

The components of NETs, including histones and neutrophil granule proteins, such as cathepsin G, MMP9, neutrophil elastase and other proteases, can have negative effects on their own tissues [73]. Vascular endothelial cells, particularly those lining the kidneys, are thought to be particularly affected by NETs, as circulating NETs can be locally deposited in them during blood filtration [74].

##### NETs and Endothelial Dysfunction

Patients with SLE have an increased risk of endothelial dysfunction and cardiovascular disease. Villanueva et al. [28] and Pieterse et al. [74] demonstrated the damaging effect of NETs from SLE patients on the endothelium in vitro [28]. NETs may be toxic to endothelial cells due to the action of granule enzymes. For example, LDGs in SLE produce NETs with increased MMP-9 content. MMP-9 of NETs activate endothelial MMP-2 and promote endothelial cell damage and apoptosis. The inhibition of MMP-9 and MMP-2 reduces NETs-induced endothelial dysfunction [41]. The beneficial effect of NETs inhibition on endothelial function has also been demonstrated in animal models. In murine models of SLE, blockade of NETs formation by a PAD4 inhibitor [36,37] has been shown to reduce endothelial dysfunction.

##### NETs in the Affected Tissues

NETs have been reported in lupus nephritis (LN) using morphological examination [28,64]. Hakkim et al. [64] reported the detection of NETs in renal biopsies of SLE patients in whom NET degradation was impaired. Villanueva et al. [28] showed that NETs were detected in the majority of kidney biopsy specimens from lupus nephritis patients. Furthermore, the percentage of glomeruli infiltrated with NETs was higher in patients with class IV LN than in patients with class III LN. Frangou et al. [35] also found NETs in renal biopsy specimens from patients with proliferative lupus nephritis. Pieterse et al. [74] found NETs in 87.5% of kidney biopsies from SLE patients, with the percentage of NET-positive glomeruli varying from 18% to 75%. The percentage of NETs-positive glomeruli correlated with the level of proteinuria and the expression of endothelial-mesenchymal transition (EndMT) markers. EndMT contributes to the transformation of the glomerular endothelium into a mesenchymal phenotype, in which cells can synthesise extracellular matrix, contributing to the development of renal fibrosis and consequently to the progression of renal failure. The same authors found that the glomeruli of MRL/lpr mice had significantly higher levels of NETs compared to healthy mice. Increased NETs in the glomeruli correlated with increased expression of EndMT markers.

The role of NETs in the development of lung fibrosis in SLE has been demonstrated. NETs were found in the affected lungs of SLE patients. In vitro, NETs from SLE patients increased the expression of EndMT markers, such as α-SMA, Twist, and Snail, and decreased the expression of E-cadherin, indicating the effect of NETs on endothelial-mesenchymal transition, including in the lungs [75].

NETs have been reported in damaged skin in SLE patients [28,35]. Villanueva et al. [28] found NETs in the dermis and subcutaneous layer of the affected skin, with a particularly high level of NETs near the dermal-epidermal junction. In another study [35], NETs were also found in skin biopsies from active discoid lupus, but not in non-inflamed skin from SLE patients or healthy individuals.

### 2.4. NETosis Markers in Peripheral Blood in SLE

Despite the accumulating data on the role of NETs in the pathogenesis of SLE in vitro, in murine models of SLE and the detection of NETs in tissue biopsy specimens from SLE patients, the clinical significance of NETs detection in patients remains poorly understood. Therefore, the study of NETs markers in peripheral blood is of particular interest.

#### 2.4.1. The MPO-DNA Complex

The myeloperoxidase deoxyribonucleic acid (MPO-DNA) complex is a specific marker of NETosis. Assaying the MPO-DNA complex in plasma and serum is the most specific, objective and quantitative way to assess NETs [76]. It is the most studied specific marker of NETs in the blood of SLE patients. All investigators [35,46,54,72,77,78] found an increase in the MPO-DNA complex in the peripheral blood of SLE patients compared to healthy controls, indicating NETosis in vivo. However, the role of the MPO-DNA complex as a biomarker for specific manifestations or activity of SLE remains controversial.

Frangou et al. [35] reported that serum MPO-DNA complex levels were significantly higher in patients with high disease activity than in patients with low disease activity or healthy controls. In a study by Bruschi et al. [46], significantly higher levels of MPO-DNA complex were found in patients with lupus nephritis. MPO-DNA complex levels positively correlated with anti-dsDNA and negatively correlated with C3 and C4. No correlation between the MPO-DNA complex and SLE activity or other clinical manifestations was found. In a study by Moore et al. [77], elevated MPO-DNA complex was associated with the presence of lupus nephritis, a history of arterial thrombosis, and a future increase in SLEDAI at 3 months and 1 year. Reshetnyak et al. [72] found that the increase in the MPO-DNA complex was associated not only with the presence of lupus nephritis but also with current SLE activity and positivity for antibodies against dsDNA and hypocomplementemia. No association between the MPO-DNA complex and other clinical manifestations or drug therapy was found. In contrast, Hanata et al. [78] found that patients with high levels of MPO-DNA complex were more likely to have pleuritis and myositis, whereas the frequency of other clinical manifestations (including lupus nephritis) or SLE activity did not differ significantly. At the same time, serum MPO-DNA complex levels were negatively correlated with anti-dsDNA and C1q-binding immune complexes. The authors suggested that their results were related to the characteristics of the cohort they studied: most patients had the “inflammatory” phenotype of SLE with fever, serositis, arthritis, myositis and, less commonly, lupus nephritis.

#### 2.4.2. Cell-Free DNA (cfDNA)

Cell-free DNA (cfDNA) is the most studied non-specific marker of NETosis. CfDNA is extracellular DNA found in serum or plasma [79]. Because the source of cfDNA can be from cells other than NETs, this marker is considered non-specific. Most investigators have found an increase in cfDNA in the peripheral blood of SLE patients [67,80,81,82,83,84]. Tug et al. [81] and Xu et al. [84] reported that significantly higher levels of cfDNA were observed in patients with active SLE. These investigators did not find an association between cfDNA and other clinical and laboratory manifestations of SLE. Abdelal et al. [83] also found that cfDNA levels were higher in patients with active SLE. They also found a correlation between cfDNA and anti-dsDNA, and C3 and C4 levels. In the study by Zhang et al. [67], the mean concentration of cfDNA was significantly higher in patients with lupus nephritis compared to patients without kidney damage. No correlation was found with other clinical and laboratory manifestations.

### 2.5. Blockade of NETs as a Therapeutic Strategy in SLE

Immune complexes and autoantibodies are triggers of NETosis in SLE. The inhibition of autoantibody synthesis via combination therapy with rituximab and belimumab reduces spontaneous NETosis in SLE patients [34]. Prevention of key factors in the development of NETs, such as PAD4 [31,36,37,48,49,50,51] and NADPH oxidase [22,31,52] in SLE, as mentioned above, provides conflicting results. The therapeutic effect of inhibition of other factors involved in NETosis in SLE, such as mitochondrial ROS [40,51,52], spleen tyrosine kinase (Syk) [47], IRE1α [40] and the JAK/STAT signalling pathway via tofacitinib [85], and autophagy [35] via hydroxychloroquine, has been shown to disrupt NET formation.

### 2.6. NETs and Thrombosis in SLE

Thrombosis in SLE and its association with NETs should be mentioned separately. It is well known that SLE is an independent risk factor for thrombosis, and the incidence of thrombotic complications in patients with SLE, even in the absence of antiphospholipid antibodies (aPL), is higher than in the average population and may be as high as 40% [86]. There is a limited number of studies on the contribution of NETs to the development of coagulopathy and thrombosis in SLE. Manzano et al. [87] investigated the relationship between thrombin generation and NETosis in patients with SLE without aPL and a history of thrombosis. They found that neutrophils from SLE patients spontaneously produced more NETs and thrombin than neutrophils from healthy controls. In a study by Frangou et al. [35], NETs from SLE patients expressed active tissue factor (TF), which promoted thrombin generation. In addition, NETs from patients with active SLE were significantly more likely to induce thrombin generation than NETs from inactive patients. In a study by Moore et al. [77], an increase in the MPO-DNA complex was associated with a history of arterial but not venous thrombosis. It is known that thrombotic complications occur predominantly during periods of high SLE activity, and NETs levels are also elevated during periods of high activity. The genesis of thrombosis may be partly due to NETosis. In a mouse model of SLE [36], the inhibition of PAD by chloramidine prevented the formation of NETs, which delayed the time to develop arterial thrombosis caused by photochemical damage. More data are available on the contribution of NETs to the development of thrombosis in antiphospholipid syndrome (APS). We present brief general views on the contribution of NETs to the development of thrombosis, and more detailed information is presented in a review by Zhou et al. [88].

## 3. NETs in Thrombosis

Thrombosis is the formation of a blood clot in the lumen of a blood vessel. For a long time, it was believed that the development of thrombosis resulted solely from a disruption of the tightly regulated balance between the procoagulant, anticoagulant and fibrinolytic systems of the blood under the influence of acquired or inherited risk factors [89]. However, this concept has been extended and it has been shown that the development of thrombosis is associated not only with disorders of the haemostasis system but also with the activation of the immune system [90]. Uncontrolled inflammation leads to the development of a prothrombotic state due to increased production of procoagulant factors, excessive activation of neutrophils, platelets and endothelial cells, and suppression of natural anticoagulants, leading to thrombosis [91].

A large number of studies have been published, demonstrating the presence of NETs in venous [92,93,94,95,96] and arterial thrombi [97,98,99,100,101] from patients and animal models of induced thrombosis. The potential effects of NETs and their components on the development of hypercoagulability have been studied [92,102,103,104,105,106,107,108,109,110,111,112,113,114,115,116]. Brief data on the prothrombotic effect of NETs are summarized in Table 1.

Most of these studies have investigated the effects of NETs components on haemostasis rather than intact NETs, the importance of which remains controversial in directly activating coagulation [102]. In addition, most studies have been conducted in vitro and it is unclear whether there are similar effects of NETs on the coagulation system in vivo.

Fuchs et al. [92] were among the first to demonstrate that intact NETs provide a platform for thrombus formation in vitro, on which prothrombotic factors such as fibrinogen, fibronectin and von Willebrand factor are concentrated, and on which platelets and erythrocytes adhere. Several components of NETs contribute to platelet activation and aggregation. For example, histones H3 and H4 have been shown to stimulate platelet aggregation [92], and Semeraro et al. [103] found that histones activate platelets by binding to Toll-like receptors (TLR) 2 and 4. An interesting discovery was the ability of histones to stimulate the expression of phosphatidylserine and FV/FVa on the platelet surface, predisposing to the formation of a “procoagulant” surface on the platelet. On the other hand, Elaskalani et al. [104] showed that NETs, independent of histones and DNA, stimulate platelet aggregation and increase the expression of phosphatidylserine and P-selectin on the platelet membrane in vitro. The authors attributed this effect to the action of cathepsin G, an important component of NETs. Another study [105] also demonstrated the ability of cathepsin G to activate platelets via protease-activated receptors 4 (PAR4 receptors). Seif et al. [106] found that cathepsin G and neutrophil elastase increased the levels of thrombospondin-1, which promotes platelet aggregation.

Several components of NETs directly or indirectly activate the coagulation cascade. For example, NETs components upregulate the expression of coagulation factor genes [107]. Oehmcke et al. [108] showed that intact NETs were able to bind and activate factor XII on their surface and trigger the contact pathway of coagulation activation in vitro. The role of histones and DNA from NETs in the formation of hypercoagulation is still under debate. It has been shown [102] that isolated histones and DNA can activate the intrinsic coagulation pathway, but not intact NETs. In contrast, Gould et al. [109] showed that intact NETs were able to activate the intrinsic coagulation pathway in a DNA-dependent manner. Wang et al. [110] found that intact NETs bound to neutrophil microparticles and the complex formed activated thrombin synthesis via the XII-dependent pathway, but not the extrinsic coagulation pathway. NETs have also been shown to participate in the activation of the extrinsic pathway of blood coagulation via the expression of tissue factor [111,112] or the induction of TF expression by endothelial cells [113]. The components of NETs were able to inhibit the function of natural anticoagulants and the fibrinolytic activity of blood. For example, cathepsin G and neutrophil elastase destroyed the tissue factor pathway inhibitor (TFPI) [114] and antithrombin III [115], and histones were able to interfere with thrombomodulin-dependent activation of protein C [116].

**Figure 2:**
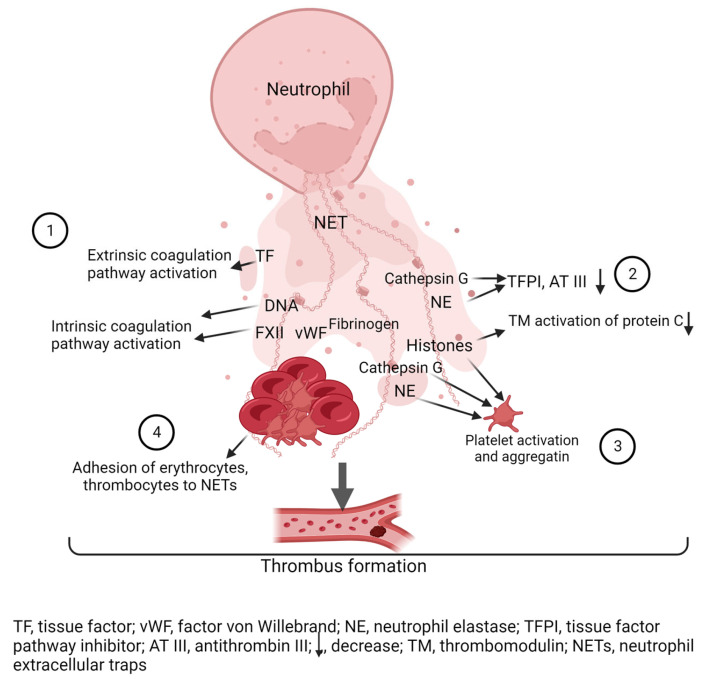
Role of NETs in thrombosis. (**1**) NETs activate the coagulation cascade: DNA NETs activate factor XII (FXII), which initiates activation of the intrinsic coagulation cascade. Tissue factor (TF) NETs activate the extrinsic coagulation pathway. (**2**) Histones, neutrophil elastase (NE) and cathepsin G inactivate anti-clotting factors: Cathepsin G and neutrophil elastase degrade tissue factor pathway inhibitor (TFPI) and antithrombin III (AT III), and histones disrupt thrombomodulin (TM)-dependent activation of protein C. (**3**) Histones, neutrophil elastase (NE) and cathepsin G activate platelets. (**4**) NETs are a scaffold for thrombus formation on which red blood cells, platelets and clotting factors concentrate. Created with BioRender.com.

Despite conflicting data, the detection of NETs in ex vivo thrombus composition, as well as the ability of components and the intact neutrophil extracellular trap itself to influence various haemostasis pathways, suggests an important role for NETs in thrombosis. Figure 2 shows a schematic representation of the contribution of NETs to the development of thrombosis.

Antiphospholipid syndrome is an autoimmune thrombophilia in which the development of thrombosis and obstetric pathology is due to circulating antiphospholipid antibodies [2]. There is increasing evidence that NETs are involved in the pathogenesis of APS [21,42,117,118,119,120,121,122,123,124,125,126,127,128,129,130,131].

## 4. NETs in APS

### 4.1. Spontaneous and Induced NETosis in APS

Yalavarthi et al. [21] showed that neutrophils from primary APS (PAPS) patients were more prone to spontaneous NET release than neutrophils from healthy donors. Isolated IgG from triple-positive APS patients stimulated control neutrophils to NETs formation significantly more than IgG from healthy donors. The authors found that antibodies against β2GPI bind to β2GPI present on the neutrophil surface and induce NETosis. Depletion of the IgG anti-β2GPI fraction inhibited NETosis, indicating a direct stimulatory effect of aPL on neutrophils. In addition, NET formation in APS was found to be dependent on NADPH oxidase and Toll-like receptor 4 (TLR4) activation. Another study [117] also showed that in vitro IgG from APS patients stimulated NETs significantly more than IgG from controls. Van der Linden et al. [42] showed that plasma from APS ± SLE patients stimulated control neutrophils to release NETs more efficiently than plasma from healthy donors. However, in this cohort, the NETs capacity in patients with APS was independent of the clinical and laboratory manifestations of the disease. Interestingly, in contrast to SLE, increased NETs formation was not associated with type I IFN activation. Zha et al. [118] showed that the anti-β2GPI/β2GPI complex induced NETs formation by neutrophils from healthy controls. As inthe study by Yalavarthi et al. [21], NETosis was mediated via the activation of NADPH oxidase and TLR4. Grossi et al. [119] showed that β2GPI binds to NETs from APS patients and the β2GPI/NET complex stimulates β2GPI-specific CD + T cells, leading to loss of tolerance, clonal expansion of β2GPI-specific B cells and aPL production. You et al. [120] also found that the anti-β2GPI/β2GPI complex induced NET formation, whereas anti-β2GPI and β2GPI alone did not stimulate NETosis. Lu et al. [121] found that neutrophils from pregnant women with PAPS were more prone to spontaneous NETosis than neutrophils from age- and gestational age-matched healthy pregnant women. The NETs formation was inhibited by blocking NADPH oxidase. In agreement with other authors, IgG from patients with APS stimulated neutrophils to netotic transformation more than IgG from controls. Li et al. [122] found that serum from APS patients stimulated neutrophils from healthy controls to release NETs significantly more than the serum from aPL carriers without clinical manifestations of APS, supporting the “double hit” theory. Ali et al. [123] showed that IgG from patients with APS stimulated NETosis in control neutrophils, in contrast to IgG from healthy donors. In contrast to previous investigators, Mauracher et al. [124] found no difference in the ability to form NETs between patients with APS and healthy controls. At the same time, as in another study [125], the levels of LDGs were significantly higher in patients with APS, but the ability to form NETs did not differ between patients and healthy controls, distinguishing APS from SLE, in which LDGs are a major source of NETs [30].

### 4.2. NETs Degradation in APS

Leffler et al. [126] observed that serum from patients with APS had a lower ability to degrade NETs than serum from healthy controls. A weak inverse correlation was found between the degree of NETs degradation and the levels of IgG antibodies to NETs (anti-NET) in APS patients. It was also found that anti-dsDNA positivity was associated with higher levels of anti-NET antibodies in patients with PAPS. Zuo et al. [127] also found impaired degradation of NETs in patients with PAPS. In this case, the presence of anti-NET IgG antibodies correlated with a low ability to degrade NETs. In addition, the depletion of IgG antibodies against NETs partially restored the ability to degrade NETs, suggesting a stabilising and protective effect of these antibodies on NETs in PAPS. Clinically, the presence of anti-NET IgG in APS patients was associated with an increased risk of recurrent venous thrombosis, and anti-NET IgM levels were inversely correlated with the levels of complement component C4. In a multinational cohort of patients [128] with aPL (of whom, 308 had PAPS), elevated levels of anti-NET antibodies were found. Anti-NET IgG and IgM correlated positively with anticardiolipin antibodies (aCL), anti-β2GP1 IgG and IgM isotypes, and antibodies to dsDNA, whereas anti-NET IgM correlated inversely with complement components C3 and C4. Anti-NET IgG positivity was associated with the presence of focal cerebral white matter lesions. In addition, anti-NET antibodies correlated directly with the MPO-DNA complex in the blood.

### 4.3. NETosis Markers in Peripheral Blood in APS

Yalavarthi et al. [21] reported increased levels of NETosis markers, such as MPO-DNA complex and cfDNA, in the plasma of APS patients compared to healthy donors. The MPO-DNA complex was significantly higher in patients with triple aPL positivity compared to patients with single positivity. Patients with a history of arterial thrombosis had significantly higher cfDNA levels. Lu et al. [121] showed that pregnant patients with obstetric APS had significantly higher serum levels of MPO-DNA complex, myeloperoxidase, neutrophil elastase and cfDNA than healthy pregnant women. Hell et al. [129] found that patients with APS and peripheral thrombosis had significantly higher levels of the specific marker citrullinated histone H3 (CitH3) than patients with isolated obstetric pathology or healthy individuals. Patients with venous thromboembolism (VTE) had significantly higher levels of CitH3 than patients with arterial thrombosis. The authors found weak direct correlations between CitH3 levels and anti-β2GPI IgG, aCL IgM and activated partial thromboplastin time (APTT). Li et al. [122] found significantly higher levels of CitH3 and cfDNA in the serum of patients with APS compared to healthy controls. Mazetto et al. [130] observed an increase in plasma MPO-DNA complex levels and increased expression of myeloperoxidase and PAD4 genes in patients with APS compared to controls, but plasma CitH3 levels and neutrophil elastase gene expression did not differ between patients and healthy controls. The authors noted an association between increased levels of MPO-DNA complex, MPO gene expression and aPL triple positivity, and between high levels of MPO-DNA complex, MPO gene expression and recurrent thrombosis. The authors concluded that elevated NETosis markers in patients with APS are associated with a more severe course of the disease. The study by Foret et al. [131] found that patients with APS who were more resistant to activated protein C (APC) had higher levels of MPO-DNA complex and cfDNA. In addition, patients who were triple positive for aPL or positive for antibodies to domain 1 of β2 glycoprotein I (aD1), i.e., patients with a high risk of thrombosis, had higher concentrations of NETs and resistance to APC than patients who were single positive for aPL or negative for aD1. In the study by Reshetnyak et al. [72], elevated levels of MPO-DNA complex were observed in 22 patients with APS (25%), and no significant association was found between high levels of MPO-DNA complex and clinical and laboratory manifestations of APS.

### 4.4. Mechanism of NETs Effect on the Hemostasis System in APS

APL-stimulated NETs have a prothrombogenic effect on the haemostasis system due to increased thrombin generation. The destruction of NETs by DNase or depletion of coagulation factors by warfarin prevented thrombin synthesis [21]. In vivo, Meng et al. [117] showed that the administration of IgG from APS patients to mice with inferior vena cava stenosis (IVS) promoted increased thrombosis compared to the administration of IgG from healthy controls. The specific marker of NETosis and CitH3 was detected at higher levels in the thrombi stimulated by aPL administration compared to controls. In addition, neutrophil depletion or DNase administration prior to IgG aPL infusion reduced the risk of thrombosis to the level of the control group. Zha et al. [118] found that aPL-stimulated NETs expressed TF and were also able to activate platelet aggregation in vitro. In vivo, in the rat carotid artery thrombosis model, the administration of the anti-β2GPI/β2GPI complex promoted accelerated carotid artery occlusion compared to the control group. Pretreatment of rats with DNase I delayed the development of thrombosis.

In APS, the interaction between neutrophils and endothelial cells via adhesion molecules is important for NETosis-mediated thrombosis [132]. For example, blockade of the adhesion molecule MAC-1 on neutrophils reduced neutrophil adhesion and NETosis in APS [133]. The deficiency of P-selectin glycoprotein ligand 1 (PSGL-1−/−), an adhesion molecule overexpressed on neutrophils in APS, resulted in reduced NETosis and thrombosis in a model of aPL-induced venous thrombosis [134]. The lower expression of Krüppel-like factor 2 (KLF2), a transcription factor that regulates and suppresses neutrophil activity, was found in patients with APS and in mice injected with aPL. KLF2 deficiency led to increased expression of PSGL-1 and TF on neutrophils and promoted greater release of NETs. PSGL-1 blockade reduced aPL-mediated thrombosis in mice [135]. In a proteomic analysis of plasma fibrin clots, Stachowicz et al. [136] found that neutrophil myeloperoxidase levels were 2.37 times higher in APS patients than in healthy controls. The authors also found that the levels of histones H2A and H2B in fibrin clots were higher in APS compared to patients with idiopathic VTE without aPL. Thus, NETs in APS have been shown to activate both plasma and platelet haemostasis, leading to vascular occlusion.

### 4.5. Blockade of NETs as a Therapeutic Strategy in APS

According to the above studies, NET formation in APS depends on the activation of NADPH oxidase and ROS [21,118], TLR4 [21,118], the activity of the adhesion molecules MAC-1 [133], and PSGL-1 [134,135], the inhibition of which suppresses NETosis. In a study by Ali et al., the activation of adenosine receptors by defibrotide [123], dipyridamole [137] and a selective A2A adenosine receptor agonist (CGS21680) [137] inhibited aPL-mediated NETosis via the suppression of ROS and reduced aPL-induced thrombosis in mice. In addition, defibrotide and dipyridamole reduced blood levels of NETosis markers in aPL-injected mice. The therapeutic effect of using DNAase to degrade NETs in APS is demonstrated in the following papers [21,117,118]. Low molecular weight heparins (LMWH) and hydroxychloroquine are drugs that are routinely used in the treatment of APS and whose mechanism of action is related to the inhibition of NETosis. These drug effects have not been studied in an aPL-stimulated NETosis, but extrapolation to APS is probably possible. For example, hydroxychloroquine inhibited NET formation [35] and LMWH not only inhibited NET formation [138] but also promoted NET destruction [92].

## 5. Conclusions

Thus, the involvement of NETs in SLE and APS has been confirmed in various in vitro, ex vivo and in vivo studies. The different effects of NETs in these two diseases may be due to the different protein composition of NETs, the action of different stimuli and the activation of different receptors. NETosis may also explain common links in the pathogenesis of SLE and APS. The question of the role of NETosis in the pathogenesis of these diseases remains open. The inhibition of NETosis as a therapeutic strategy for the treatment of SLE and APS is of great interest. Further studies are needed.

## Figures and Tables

**Table 1:** Prothrombotic effect of neutrophil extracellular traps and its components.

Authors	NET Components /Intact NET	Results	Impact on Hemostasis
Fuchs et al. [92]	Intact NET	Adhesion, activation and aggregation of platelets	-Platelet activation -NETs provide a scaffold for platelets and RBCs adhesion.
Elaskalani et al. [104]	Intact NET	Platelet activation and aggregation	Platelet activation
Sambrano et al. [105]	Cathepsin G	PAR4-dependent platelet activation	Platelet activation
Seif. et al. [106]	Neutrophil elastase, cathepsin G	Induction of thrombospondin-1 proteolysis	Platelet activation
Noubouossie et al. [102]	DNA, histones, intact NET	DNA and histones activated thrombin generation	Activation of coagulation by DNA and histones but not intact NETs
Semeraro et al. [103]	Histones	-Adhesion, activation and aggregation of platelets -Stimulation of platelet-dependent thrombin generation	-Platelet activation -Activation of coagulation
Gould et al. [109]	Intact NETs	DNA-dependent thrombin generation	Activation of coagulation
Wang et al. [110]	Intact NET	Factor XII activation and thrombin formation	Activation of coagulation
Oehmcke et al. [108]	Intact NET	Activation of factor XII	Activation of coagulation
Kambas et al. [111]	Intact NET	TF expressing NETs	Activation of coagulation
Stakos et al. [112]	Intact NET	TF expressing NETs	Activation of coagulation
Folco et al. [113]	Intact NET	NETs induce endothelial TF expression	Activation of coagulation
Petersen et al. [114]	Neutrophil elastase and cathepsin G	Inactivation of tissue factor pathway inhibitor (TFPI).	Inhibition of the natural anticoagulants
Jordan et al. [115]	Neutrophil elastase	Inhibition of antithrombin III	Inhibition of the natural anticoagulants
Ammollo et al. [116]	Histones	Disruption of thrombomodulin-dependent protein C activation	Inhibition of the natural anticoagulants

**Note:** NETs, neutrophil extracellular traps; RBCs, red blood cells; PAR4, protease-activated receptors 4.

## Data Availability

Not applicable.
